# Autonomic regulation in muscular dystrophy

**DOI:** 10.3389/fphys.2013.00257

**Published:** 2013-09-20

**Authors:** Corrado Angelini, Rita Di Leo, Paola Cudia

**Affiliations:** Fondazione IRCCS San Camillo HospitalVenice, Italy

**Keywords:** myotonic dystrophy, autonomic nervous system, heart rate, dystrophin, congenital disorders of glycosylation

Autonomic Nervous System (ANS) function has been assessed in several muscular dystrophies. In the last two decades it has been recognized a significant relationship between ANS and cardiac mortality (Politano et al., 2008). Cardiovascular ANS is frequently studied by using cardiovascular autonomic reflex tests and spectral analysis of heart rate in time and frequency domain (Ewing and Clarke, 1982; van Ravenswaaij-Arts et al., 1993). Heart rate variability (HRV) analysis, which involves measurement and analysis of heart rate variation, provides a quantitative marker of autonomic activity and has proved to be able to detect and measure modification in sympathetic or vagal activity. Moreover, it can be used to estimate the susceptibility for heart arrhythmias (Heart rate variability, 1996a,b).

## Dystrophinopathies

Duchenne (DMD) and Becker (BMD) muscular dystrophies are X-linked recessive disorders due to complete or partial loss of dystrophin protein. The presence of persistent sinus tachycardia, atrial and ventricular arrhythmias, sweating and chills has suggested an ANS involvement in these disorders.

Yotsukura et al. first described an impairment of ANS in 55 DMD, characterized by an increase in sympathetic activity and a reduction in parasympathetic output. These abnormalities were present early in the course of the disease, when cardiopulmonary function was still essentially normal, and became more marked with the progression of the disorder. The authors demonstrated also that, although HRV tended to decrease with the progress of clinical severity, it cannot be considered a good predictor of death in DMD (Yotsukura et al., 1995, 1998).

ANS impairment was confirmed by Lanza et al. (2001) and by Inoue et al. (2009) who proposed that the mean heart rate during night could be used as a simple measurement for evaluation of autonomic function and that early treatment against tachycardia should be initiated when a patient's mean heart rate at night is > 71 beats/min.

The neuropathological evidence of ANS involvement in DMD has been shown in mdx mice, where a selective loss of the sympathetic superior cervical ganglion (SCG) neurons, combined with the dystrophin-associated muscle damage, might lead to ganglion neuron death (De Stefano et al., 2005).

In 1997, the arrhythmic profile in a population of 20 BMD patients was investigated to correlate the severity of arrhythmic events, the cardiac autonomic balance (assessed by HRV analysis in the time domain) and the degree of left systolic impairment (Ducceschi et al., 1997). In this study they observed a cardiac autonomic imbalance characterized by sympathetic predominance and an increased susceptibility to ventricular arrhythmias, even in the absence of overt cardiomyopathy; however, the severity of arrhythmic profile was closely related to the degree of left ventricular systolic dysfunction (Ducceschi et al., 1997).

In a subsequent study, 20 BMD patients were investigated with a battery of six cardiovascular autonomic tests and power spectral analysis of HRV (Vita et al., 2001). Although 11 patients revealed abnormal findings at some cardiovascular tests, none of them had a definite autonomic damage, as indicated by two or more abnormal tests. This clinical observation suggested therefore that autonomic involvement does not represent a major finding in BMD (Vita et al., 2001).

In 2006 the prognostic value of HRV for sudden death was evaluated in a population of 30 BMD patients and dilated cardiomyopathy compared with a control group of 30 normal subjects (Ammendola et al., 2006). An increment of sympathetic tone in BMD was observed, characterized by a lower HRV in the frequency and time domains and a higher mean heart rate than in the control group. HRV was lower in the high arrhythmic risk BMD group than in the remaining patients. All BMD patients who died had lower HRV than the minimum HRV recorded in the survivors and none of them had an abnormal standard deviation of all normal RR interval over 24 h (SDNN). SDNN is considered the most important parameter to estimate the adrenergic activity, being inversely related to it. This study concluded that ANS may have an important role in BMD and that SDNN values <100 ms may be a significant predictors of cardiac death, independent of clinical variables (Ammendola et al., 2006). No study has been so far conducted in DMD/BMD carriers.

## Myotonic dystrophies

Myotonic dystrophy (DM) is a chronic and slowly progressive, autosomal-dominant, multisystem disease characterized by a wide spectrum of clinical findings and by marked intrafamilial and interfamilial clinical variability. There are two genetically distinct form of DM, myotonic dystrophy type 1 (DM1) and type 2 (DM2) with different clinical phenotypes. The most frequent form is DM1 and on the basis of clinical severity the disorder is divided into three groups: mild, classical, and congenital. DM1 is caused by a (CTG)n expansion in the gene encoding Dystrophia-Myotonica-Protein-Kinase (DMPK), a zinc finger protein. When transcribed into CUG-containing RNA, mutant transcripts aggregate as nuclear foci that sequester RNA-binding proteins, resulting in a spliceopathy of downstream effector proteins (troponin, insulin receptor, etc.). DM1 may be considered as a toxic RNA disease (Udd and Krahe, 2012; Kumar et al., 2013).

In DM1 symptoms suggestive of ANS involvement such as heart rhythm conduction disturbances, orthostatic hypotension, disorders of sweating, abnormal gut movements, and dysfunction of genitourinary apparatus are frequently referred (Aminoff et al., 1985). Conduction and heart rhythm disturbances are characterized by brady-arrhythmias leading to atrioventricular block, ectopic beats, atrial fibrillation, flutter, and other types of tachy-arrhythmias. We found a correlation between conduction heart system defects and (CTG)n expansion (Melacini et al., 1995). A systematic heart function follow up should be performed in DM1 patients periodically, even in asymptomatic patients to prevent sudden death.

Cardiovascular ANS is frequently studied by using cardiovascular autonomic reflex tests and spectral analysis of heart rate in time and frequency domain (Ewing and Clarke, 1982; van Ravenswaaij-Arts et al., 1993).

The cardiovascular autonomic function test was first investigated in DM1 demonstrating only minor signs of parasympathetic dysfunction and suggested that the symptoms referred to an autonomic dysfunction could be ascribed to target organ abnormality rather than to ANS dysfunction (Olofsson et al., 1990; den Heijer et al., 1991; Pierangeli et al., 1992).

In 23 DM1 patients a battery of cardiovascular reflex tests were studied, and in parallel nerve conduction, heart and breathing evaluation were done. Fourteen patients showed some alterations and one a definite autonomic damage to the battery of cardiovascular tests without correlation to other clinical parameters including heart abnormality, electro-neurography or spirometry (Di Leo et al., 2004). Subsequently, a battery of autonomic tests was performed in 20 DM1 patients and the same sample sympathetic and vagal hyper/hypoactivity were observed (Rakoèević-Stojanović et al., 2007). The limits of these studies are the small size of DM1 patient cohorts and the different clinical techniques employed.

HRV and its indices in time and frequency domain may be considered as indicators of autonomic modulation of the heart and a possible predictor of mortality for sudden cardiac death or fatal arrhythmias in a series of different heart conditions such as congestive heart failure, ischemic heart disease or after heart transplantation. HRV study was also investigated in various types of muscular dystrophies, including DM1 (Politano et al., 2008), to predict sudden cardiac death.

The total power and low-frequency component (a marker of sympathetic and vagal modulation of heart rate) and high-frequency component (a marker of vagal modulation of heart rate) resulted to be lower than those in the control group (Inoue et al., 1995).

Hardin et al. (2003) studied HRV in 289 DM1 patients showing that the 24-h time domain parameters of SDNN and SDANN decrease with age and with increased (CTG)n repeat length (Hardin et al., 2003).

Di Leo et al. (2004) found that a reduction in the supine low-frequency band inversely correlates with disease duration and low-frequency power during standing and was significantly associated with presence of heart involvement (Di Leo et al., 2004). Both 24-h time domain parameters of SDNN and total power resulted significantly lower in DM1 patients than in healthy controls (Rakoèević-Stojanović et al., 2007). Magrì et al. (2012) recently studied in 43 DM1 patients the QT variability index as a marker of temporal myocardial repolarization beyond cardiac conduction abnormalities and power spectral components. The first one resulted higher while the second ones lower in DM1 than normal controls (Magrì et al., 2012).

Fregonezi et al. (2012) found influence of gender and disease on HRV in DM1 patients and healthy individuals in different body positions (sitting and supine). HRV was done during short time periods in sitting and supine positions in addition to analyse the magnitude of gender influence. They found that males with DM1 have a different behavior than women both in sitting and standing position. This study is important in several ways since it indicates a gender difference in sympathetic dysfunction in DM1.

## Facio scapulo humeral muscular dystrophy

Facio Scapulo Humeral Muscular Dystrophy (FSHD) is the third most frequent form of muscular dystrophy. HRV was evaluated in 55 FSHD patients and evidenced the presence of autonomic modifications, characterized by slight increase in sympathetic activity and a progressive decrease in parasympathetic output, which became more evident with the progression of the disease (Della Marca et al., 2010).

## Emery-dreifuss muscular dystrophy

Emery-Dreifuss Muscular Dystrophy (EDMD) is genetically heterogeneous since it may be caused by a mutation in the STA gene encoding emerin (X-linked inheritance) or as AD trait determined by mutations in the LMNA gene encoding lamin A/C. EDMD is clinically characterized by a slow progressive muscular dystrophy, joint contractures and prominent cardiomyopathy with cardiac conduction abnormalities that might cause sudden death.

Fujita et al. performed I ^[123]^ MIBG myocardial scintigraphy in a patient with a nonsense mutation in the STA gene. MIBG is an analog of norepinephrine and is stored in presynaptic terminals of sympathetic nerves. The scintigraphy evidenced a diffuse and severe decrease in accumulation of MIBG in the heart suggesting an abnormality in the cardiac sympathetic nerve terminals in the patient. They suggested that the abnormality on I ^[123]^ MIBG myocardial scintigraphy may predict LV dysfunction in EDMD (Fujiita et al., 2005).

## Miyoshi muscular dystrophy

Miyoshi muscular dystrophy (MMD) is caused by mutation in the gene encoding the protein dysferlin. In 1994, Tomoda et al. performed accurate autonomic studies in 2 MMD affected girls, including laser doppler flowmetry, study of the component analysis of the cardiographic R-R interval and sympathetic skin response (SSR). They demonstrated marked abnormalities such as sensitive vasoconstrictive response, a suppressed peak of low frequency components, and an absence of SSR, compared to healthy controls. Sympathetic nerve blocking produced clinical improvement (Tomoda et al., 1994).

## Fukuyama congenital muscular dystrophy

Fukuyama-type congenital muscular dystrophy (FCMD) is an autosomal recessive disease caused by mutation in the fukutin gene. The study of brainstem tissue of 10 patients affected by FCMD showed a notable reduction of the catecholaminergic neurons in the reticular formation, vagal nuclei, and nucleus tractus solitarius of FCMD cases compared to controls. The Authors suggested that neuronal dysfunction in the cardiovascular and respiratory centers may lead to sudden death in FCMD (Itoh et al., 1996).

In conclusion, in agreement with ENMC International Workshop (van Engelen et al., 2005) our suggestion for autonomic dysfunction in muscular dystrophy is presented in Table 1. In particular, in DM1, cardiac problems can be unrecognized. Since physical activities precipitate arrhythmias, we advise periodic monitoring in DM1, dystrophinopathies, and EDMD. A program of HRV monitoring should be done before exercise and rehabilitation in patients with overt autonomic dysfunction.

**Table 1:** **Autonomic dysfunctions in various types of muscular dystrophies**.

**Type of dystrophy**	**Severity of ANS involvement**	**Complication needing prevention or treatment**
Duchenne muscular dystrophy (DMD)	++	Tachycardia, atrial and ventricular arrhythmia, sweating, and chills
Becker muscular dystrophy (BMD)	+	Lower HRV in frequency in high arrhythmic BMD, increased sympathetic tone
DM1	++	Orthostatic hypotension, heart rhythm disturbances, abnormal gut movements, bradi-arrhythmia leading to heart block
EDMD	++	Heart block (defibrillator)
FSHD	±	Tachycardia
Miyoshi myopathy	±	Abnormal sympathetic skin response and vasoconstrictive response
FCMD	+++	Brain stem dysfunction, reduction of cathecolaminergic neurons in reticular formation, and nucleus tractus solitarius
